# Peptide- and Metabolite-Based Hydrogels: Minimalistic Approach for the Identification and Characterization of Gelating Building Blocks

**DOI:** 10.3390/ijms241210330

**Published:** 2023-06-19

**Authors:** Om Shanker Tiwari, Sigal Rencus-Lazar, Ehud Gazit

**Affiliations:** 1 The Shmunis School of Biomedicine and Cancer Research, George S. Wise Faculty of Life Sciences, Tel Aviv University, Tel Aviv 6997801, Israel; otiwari1992@gmail.com (O.S.T.); sigal.lazar@gmail.com (S.R.-L.); 2 Department of Materials Science and Engineering, The Iby and Aladar Fleischman Faculty of Engineering, Tel Aviv University, Tel Aviv 6997801, Israel; 3 Sagol School of Neuroscience, Tel Aviv University, Tel Aviv 6997801, Israel

**Keywords:** peptide, metabolite, supramolecular hydrogel, amino acids, drug delivery, tissue regeneration, tissue engineering, and biomedical applications

## Abstract

Minimalistic peptide- and metabolite-based supramolecular hydrogels have great potential relative to traditional polymeric hydrogels in various biomedical and technological applications. Advantages such as remarkable biodegradability, high water content, favorable mechanical properties, biocompatibility, self-healing, synthetic feasibility, low cost, easy design, biological function, remarkable injectability, and multi-responsiveness to external stimuli make supramolecular hydrogels promising candidates for drug delivery, tissue engineering, tissue regeneration, and wound healing. Non-covalent interactions such as hydrogen bonding, hydrophobic interactions, electrostatic interactions, and π–π stacking interactions play key roles in the formation of peptide- and metabolite-containing low-molecular-weight hydrogels. Peptide- and metabolite-based hydrogels display shear-thinning and immediate recovery behavior due to the involvement of weak non-covalent interactions, making them supreme models for the delivery of drug molecules. In the areas of regenerative medicine, tissue engineering, pre-clinical evaluation, and numerous other biomedical applications, peptide- and metabolite-based hydrogelators with rationally designed architectures have intriguing uses. In this review, we summarize the recent advancements in the field of peptide- and metabolite-based hydrogels, including their modifications using a minimalistic building-blocks approach for various applications.

## 1. Introduction

Hydrogels are composed of molecular networks that can retain a substantial amount of water inside their structures (in most cases, water comprises most of their mass). These assemblies retain a solid structure and do not dissolve in water. Naturally occurring hydrogels include collagen and gelatin. Various synthetic molecular and supramolecular polymers have been shown to form hydrogels. The conventional hydrogel networks are produced during the initial stages of hydrogel growth using covalently cross-linked polymers such as poly(ethylene glycol) diacrylate (PEGDA), poly(ethylene glycol) monoacrylate (PEGMA), poly(ethylene glycol), poly(vinyl alcohol), polyacrylamide, and chitosan-collagen [1,2,3,4,5,6]. These inexpensive chemically cross-linked hydrogels have limited applicability in the field of regenerative medicine and other biomedical applications due to their high molecular weight and potentially toxic chemicals or catalysts, which require prudent inspection of the safety profile [7]. The foundations of bio-inspired materials design with an emphasis on the advantages and limitations of decellularized and reconstituted biopolymeric matrices as well as biohybrid and fully synthetic polymer hydrogel systems have been applied to enable specific organotypic and organoid cultures [8]. Furthermore, there are emerging adhesive qualities of soft and hydrated surfaces for the design of binding chemistry or adhesion junctions, whether covalent, dynamic covalent, supramolecular, or physical [9]. For a variety of biomedical applications, wet adhesion is helpful for adhering to or between tissues and implants. In the context of effective junction design, a number of recent and upcoming adhesive hydrogels for use in biomedicine have been reported. The major objective is to control the adhesion strength, reversibility, stability, and sensitivity to environmental stimuli by engineering hydrogel adhesion through the molecular design of the junctions.

Moreover, due to their highly structured assembly patterns, biological origins, bioactivity, easy synthesis, characterization, chemical diversity, high stability, biocompatibility, low cost, and biodegradability, peptide- and metabolite-based materials, which are present everywhere in nature, are able to carry out specialized biological tasks. Self-assembly through noncovalent interactions such as electrostatic, hydrophobic, and hydrogen bonding, as well as π–π stacking interactions, can mediate a variety of supramolecular nanoarchitectures, including nanotubes, nanoparticles, nanofibers, nanobelts, and hydrogels [10,11,12,13,14,15,16,17,18]. Non-covalent interactions, which are often weaker than covalent bond interactions and allow supramolecular gelators to self-assemble into hydrogels, are primarily responsible for this phenomenon [19]. Amino acids and their derivatives are a family of supramolecular gelators, and the fundamental mechanism underlying their gelation is intermolecular hydrogen bonding between amide bonds [20,21,22,23]. With a focus on understanding the relationships between their structures, properties, and functions, the current progress in the preparation of peptide and amino acids-based hydrogels under various types of external stimuli, as well as the in situ encapsulation of cells into the hydrogels have been reported [24]. The applications of peptide- and amino-acid-based hydrogelators with rationally chosen architectures in regenerative medicine, tissue engineering, and pre-clinical testing seem promising. Furthermore, the development of biomacromolecules, including peptide-based multifunctional hydrogels with a combination of various characteristics and functions, such as antioxidant, antibacterial, bioadhesive, and sustaining mechanical properties, as well as their effect on wound healing, seems to offer similar promise [25]. The peptide-based multi-responsive hydrogels with unique and improved properties using coarse-grained and atomistic simulations can be useful in exosome delivery, with an emphasis on bioadhesion, organoids, and tissue engineering [26]. The remarkable properties of peptide- and metabolite-based hydrogels suggest their potential use in 3D bioprinting, tissue engineering, antibacterial and wound-healing materials, drug delivery, anti-bacterial, tumor therapy, tissue engineering, water remediation, and other biomedical applications [27,28,29,30,31,32,33,34,35,36,37,38,39,40,41,42,43,44,45,46,47,48,49,50,51,52,53,54,55,56,57,58,59,60,61,62,63,64,65,66]. 

Here, we summarize the latest development of peptide- and metabolite-based hydrogels, including their biomedical and technological applications. In particular, we discuss the superior injectability, stiffness, and stability of numerous hydrogels based on peptides and metabolites for biomedical applications. We primarily focus on the recent development of reliable peptide- and metabolite-based hydrogel fabrication strategies and applications in the biomedical field. Designing hydrogels with tunable structures and enhanced mechanical stability is important for their biological applications. Moreover, by modifying the peptides and metabolites with various amino acid types, sequences, and other alterations, it is possible to alter the structural and functional features of these hydrogelators. The biomedical uses of hydrogels in the areas of drug delivery, anticancer therapy, antibacterial, wound healing materials, 3D bioprinting, and tissue engineering are then highlighted. 

## 2. Peptide-Based Hydrogels

**Figure 1:**
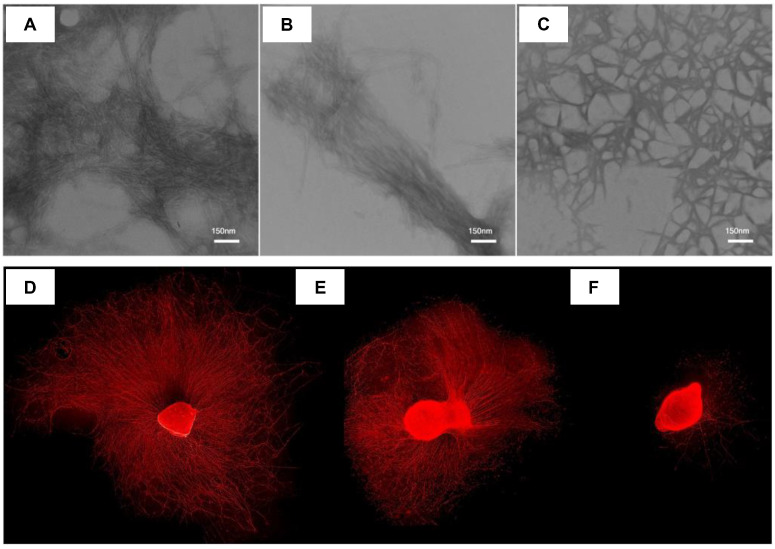
(**A**) The nanofibrous structure of the hydrogel as observed by TEM images of the IKVAV–PA hydrogel following negative staining with 2% uranyl acetate. (**B**) Alignment of the nanofibrous structure triggered by the inclusion of BDNF. (**C**) The porosity of the IKVAV–PA hydrogel was increased by degradation over the course of 21 days of hydrogel incubation under physiological conditions. (**D**,**E**) Neurite outgrowth from DRG explants stimulated by BDNF (**D**) released from the IKVAV–PA hydrogel and (**E**) present in the solution at the same concentration. (**F**) Significantly reduced DRG explant neurite outgrowth was observed in the presence of aCSF alone in the cell culture medium. Reproduced with permission from Ref. [68]. Copyright © 2019, Hassannejad et al., *Injury*, Published by Elsevier Ltd. All rights reserved.

The self-assembled peptide hydrogel scaffold has a number of advantages over polymeric hydrogels, such as a nanofibrillar structure, tunable mechanical properties, and suitable biodegradability, which enables the formation, growth, and natural release of spheroids with a controllable and uniform size of up to 1 mm for the purposes of 3D bioprinting, tissue engineering, and organ regeneration. As shown by Raphael et al., the self-assembled peptide-based hydrogels can be used for 3-dimensional printing with the encapsulation of epithelial cells [67]. Furthermore, the distinct architectures and enhanced structural integrity resulted in the possibility of systematic printing of the three-dimensional matrices. It was further confirmed that mammary epithelial cells remained viable and began to proliferate after 7 days of culture, independent of the stiffness of the hydrogel, which is useful for the modulation of cellular responses. To produce a favorable environment for cell migration and growth, as well as the sustained release of brain-derived neurotrophic factor (BDNF) at the lesion following severe compression injury, Hassannejad et al. synthesized an isoleucine–lysine–valine–alanine–valine (IKVAV)-functionalized peptide amphiphile (PA) hydrogel [68]. The nanofibrous supramolecular structure of the IKVAV–PA hydrogel before and after incorporation of BDNF within the hydrogel network was visualized by transmission electron microscopy (TEM), showing BDNF incorporation within the hydrogel network, where the nanofibers organized as aligned structures (Figure 1A,B). Furthermore, the pore size and porosity of the BDNF-loaded IKVAV–PA hydrogel network were increased after 21 days of incubation within the artificial cerebrospinal fluid (aCSF) at 37 °C (Figure 1C). The authors examined and compared the released BDNF’s capacity to promote neurite outgrowth from dorsal root ganglion (DRG) explants to that of aCSF as a control. DRG explants were exposed to the released BDNF and hydrogel breakdown products over the course of 48 h, despite not coming in direct contact with the hydrogel. The degree of neurite outgrowth from the DRG explants was tested by immunostaining against ß III tubulins (Figure 1D–F). Although locomotor functional recovery was not examined/observed in this study, the axon preservation and the low levels of inflammation detected in animals treated with a BDNF-containing hydrogel suggest that the designed intervention has the potential to be tested further in the quest to produce effective treatments for severe spinal cord injury.

Furthermore, Park et al. have reported that when an active RGD peptide hydrogel was added to hyaluronic acid (HA), cell adhesion and proliferation changed dramatically [69]. Cells cultivated on the RGD-modified hydrogel proliferated and expanded to confluence, but cells cultured on the RDG control hydrogel showed, for long periods of time, no indication of spreading in culture or in the presence of serum proteins. In contrast, cells cultivated on the RGD–HA hydrogel multiplied and expanded to confluence. Alheib et al. presented a skeletal muscle platform using myoblasts that were differentiated and aligned on hydrogels composed of micropatterned gellan gum, which was then customized with a peptide derived from laminin [70]. Peptide binding experiments using murine skeletal muscle cells (C2C12) demonstrated that C2C12 could bind the laminin-derived peptides RKRLQVQLSIRTC (Q), KNRLTIELEVRTC (T), and CIKVAVS (V) based on peptide-receptor affinity. This adult skeletal muscle surrogate has the potential to be a powerful tool for drug screening applications as well as a platform for research into the physiology and pathophysiology of skeletal muscles.

Gyenes et al. showed the swelling properties of aspartic-acid-based hydrogels in which the mechanism is largely dependent on the pH of the swelling solution, as demonstrated by a comparison of the swelling kinetics of polysuccinimide (PSI)- and poly(aspartic acid) (PASP)-based gels in an alkaline medium. These hydrogels have been suggested for use as dispersing agents in commercial applications [71]. The cooperative diffusion constant of swelling was equivalent for PSI- and PASP-based gels at pH 8, where solvent exchange, hydrolysis, and swelling happened sequentially in three separate reactions. Pasc et al. synthesized and described a β-Ala-His-EO_2_-Alk peptide-based hydrogel [72]. As a result of close contacts between gelator molecules, the number of amide groups controlled the hydrogel’s ability to self-assemble into 1D or 2D/3D networks. Furthermore, Chen et al. synthesized two short amphiphilic peptides, Ac-I_3_SLKG-NH_2_ and Ac-I_3_SLGK-NH_2_. It was shown that these two peptides self-assembled in aqueous solutions to produce hydrogels with quick shear-thinning and recovery capabilities [73]. The Ac-I_3_SLKG-NH_2_ hydrogel showed considerable enzymatic vulnerability, with MMP-2 severely impairing secondary structure formation, the nanofiber network, and rheological properties. The self-assembled nanofibers and the entangled network were considerably disrupted by MMP-2 treatment, resulting in a steady decrease in the mechanical properties of Ac-I_3_SLKG-NH_2_ hydrogels, indicating susceptibility to enzymatic cleavage. Moreover, when an anticancer peptide was physically entrapped in the hydrogel in the presence of HeLa cells, the drug release was found to be regulated by the MMP-2 levels, resulting in significantly suppressed cancer cell growth. Together, these unique qualities of the Ac-I_3_SLKG-NH_2_ hydrogel allow for a number of biomedical applications, including regulated drug delivery and regenerative therapy, making them appropriate as smart materials. Braun et al. described the identification of the microscopic processes underlying the self-assembly of SgI_37–49_, a peptide hydrogelator [74]. Furthermore, SgI_37–49_ fibril formation was fueled by fibril-catalyzed secondary nucleation, and all microscopic processes involved in SgI_37–49_ self-assembly exhibited an enzyme-like saturation behavior by using theoretical models of linear polymerization to analyze the kinetic self-assembly data. The authors showed a novel mechanistic methodology for the investigation of self-assembling hydrogel-forming peptides that are complementary to established characterization methods in materials science. Oyen et al. evaluated a peptide hydrogel composed of the amphipathic hexapeptide H-FEFQFK-NH_2_ for its controlled release qualities and its stability by in vivo imaging [75]. Zhu et al. synthesized three different types of short cationic peptides (RGDK, RRRFK, and RRRFRGDK) combined with poly(ester amide) (PEA)-based hydrogel to produce a series of amino-acid-derived pseudo-proteins [76]. Direct conjugation of the RRRFRGDK peptide to the hydrogel surface via an amidization reaction improved its antibacterial and hemostatic properties with little to no impact on the original hydrogels’ outer appearance and internal morphology. The peptide-functionalized hydrogel showed high mechanical strength, good hemocompatibility and cytocompatibility, enzymatic biodegradation, and high water-uptake capacity. The findings suggest that peptide-functionalized PEA-based hydrogels can be employed as hemostasis agents and wound dressings for infected wounds.

Bairagi et al. showed that a transparent hydrogel composed of histidine (His)-containing peptides can be self-assembled in phosphate-buffered saline (PBS) at pH 7.5 [77]. This study also persuasively demonstrated how adding various dicarboxylic acids to the system can modify the mechanical stiffness and thermal stability of the peptide-based supramolecular hydrogel, enhancing hydrogen bonding and electrostatic interactions within the gel phase. Additionally, peptide-gel-based soft biomaterials have the potential to be used in the future in regenerative medicine due to their tunable mechanical strength, thermal stability, and hydrogel structures. Yadav et al. described a tetrapeptide (Asp-Leu-IIe-IIe), the shortest peptide sequence from the highly fibrillogenic protein TAR DNA-binding protein 43 (TDP-43), which self-assembled into a hydrogel [78]. The hydrogel had a storage modulus in the MPa range and was extremely stable and mechanically robust. The ultrashort peptide-based hydrogel presented in this study has significant potential for future development as an engineering scaffold and/or a tissue regeneration tool. Thota et al. combined a dipeptide hydrogelator and a short chemotactic peptide ligand that attracts macrophages, thereby producing an injectable, ultrashort bioactive peptide hydrogel [79]. The skin antibiotic ciprofloxacin, among others, was shown to be slowly and continuously released by the hydrogels. Such bioinspired advanced functional materials can be used to treat chronic wounds such as diabetic foot ulcers.

Furthermore, Peltier et al. reported the first instance of a peptide-based hydrogel that responds to hydrogen sulfide (H_2_S) [80]. The functionalization of an ultrashort hydrogelating peptide sequence with an azidobenzyl moiety, which was reported to preferentially react with H_2_S under physiological conditions, was the basis of the peptide-based hydrogel design. Under various physiological and pathological conditions, the concentration of H_2_S in living biosystems changes in the nanomolar-to-millimolar range. At concentrations as low as 0.1 wt%, the resultant ultrashort peptide can produce hydrogels that could be used in various biomedical applications. The authors believe that the innovative hydrogel produced in this study may offer practical resources for biomedical research. Kulkarni et al. described a new class of hydrogelators based on helical β^3^-peptides containing a bioactive payload [81]. The β^3^-peptides self-assembled in an aqueous solution to produce a stable hydrogel by forming nanofibrous supramolecular structures. Conte et al. showed that, upon sonication of peptide solutions, the well-known self-assembling dipeptide diphenylalanine (Phe-Phe) and its amidated derivative (Phe-Phe-NH_2_) can produce metastable hydrogels [82]. Upon mechanical contact, the hydrogels exhibited instantaneous syneresis, which caused a rapid evacuation of water, followed by their collapse into a semi-solid gel. Hydrogels of this type, based on a simplistic dipeptide building-block, can be used as pressure sensors or as selective scavengers for small hydrophobic molecules due to their sensitivity to external mechanical stimuli, rapid supramolecular collapse, and the extremely hydrophobic property of the fibers produced. According to Yin et al., tanshinones IIA and whole tanshinone extract were both chosen to be delivered using a unique drug-delivery system composed of a readily self-assembled and gelated octa-peptide, FHFDFHFD [83]. The drug delivery system demonstrated improved anticancer capability, sustained drug release, and enhanced loading capacity. Furthermore, Palui et al. showed that luminescent CdS nanoparticles were immobilized by three synthetic tripeptides that self-assembled in an aqueous medium in a pH-dependent manner [84]. When these peptides produced a gel in an aqueous medium at basic pH levels (pH 11.0–13.0), they formed a nanofibrillar network structure, and the production of CdS nanoparticles on the gel nanofiber resulted in a luminous property. According to this research, the optoelectronic characteristics of CdS nanoparticles can be modified without changing the size of the particles by depositing them on gel nanofibers.

**Figure 2:**
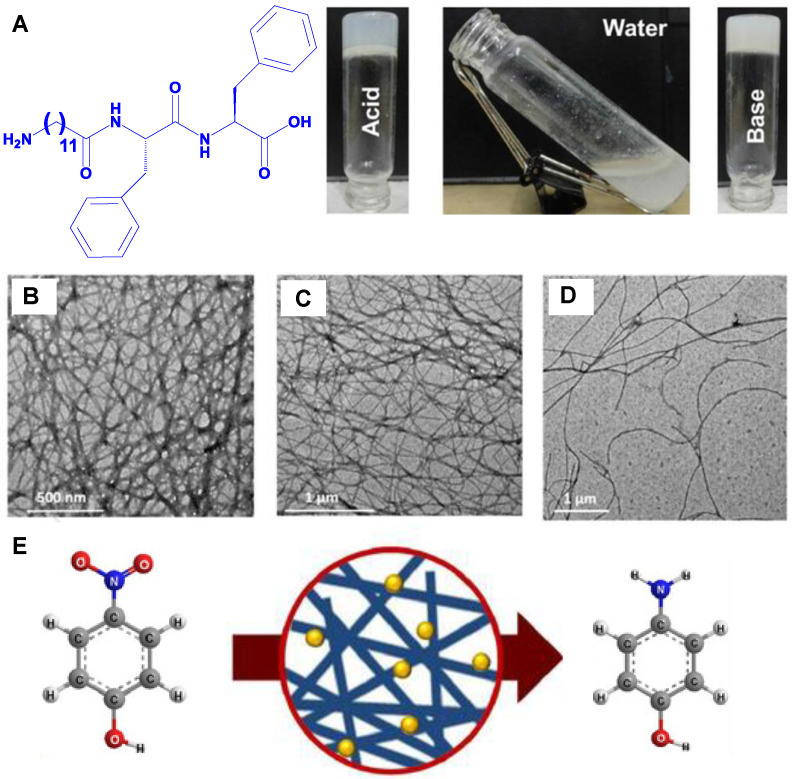
(**A**) Soluble aggregate made from the self-assembling peptide in Milli-Q water at pH 6.7, along with hydrogels made from the peptide in the presence of 0.05 M HCl (acid) and 0.05 M NaOH (base). (**B**–**D**) FEG–TEM images of the hydrogel in acid, basic, and water, respectively. (**E**) The transformation of p-nitrophenol into p-aminophenol using the hybrid hydrogel containing peptide and silver nanoparticles. Reproduced with permission from Ref. [86]. Copyright © 2018, Paul et al., *ChemNanoMat*, Published by John Wiley and Sons. All rights reserved.

Jo et al. reported that tri-amino acid peptides with two cysteine residues for crosslinking on either side of lysine (or arginine) worked well as minimal plasmin-sensitive peptides in hydrogels composed of poly(ethylene glycol) based on Michael-type addition [85]. Paul et al. reported that at the two pH extremes (in the presence of an acid or a base), a tripeptide with an oligo-methylene group was discovered to produce hydrogels; however, at neutral pH, it did not (Figure 2A) [86]. The peptide exhibited dense nano-fibrillar network structure in the cases of acidic (0.05 M HCl) (Figure 2B) and basic solutions (0.05 M NaOH) (Figure 2C) but it produced soluble aggregates in water at pH 6.7 (Figure 2D), which was confirmed using the field emission gun transmission electron microscope (FEG-TEM). Moreover, it is interesting to note that the transformation of p-nitrophenol into p-aminophenol and p-nitroaniline into p-phenylenediamine may be successfully accomplished using the hybrid hydrogel containing silver nanoparticles (Figure 2E). It is also noteworthy that the native hydrogel could not function as a catalyst when free of Ag nanoparticles. The fact that this hybrid hydrogel has been reused a few times without significantly losing its functionality also demonstrates the potential of the new nanohybrid system for catalysis applications.

Furthermore, peptide gelators composed of various chiral amino acids exhibited radically distinct hydrogel behavior and rheological features for self-assembled peptide hydrogels that might be useful in various biomedical applications [87,88,89,90]. Li et al. showed that by adjusting the concentration of oxidized glutathione (OGH) at a constant concentration of sodium deoxycholate (SD), hydrogels with various microstructures, such as nanoribbons, rigid nanofibers, and helical ribbons, could be produced [91]. A fundamental bilayer unit made up of OGH and SD molecules was stabilized by hydrogen bonds, electrostatic interactions, and hydrophobic interactions. The fundamental bilayer unit was made up of β-sheets and random coil structures. Nanoribbons and nanofibers were formed at lower OGH concentrations. However, the formation of β-sheets at higher OGH concentrations was essential for the development of chiral helical ribbon architectures. The twisting force increased as the helical ribbons grew, which might eventually cause the edges of the helical ribbons to merge and form nanotubes. These “weak hydrogels” showed an irreversible response to heating, since the secondary structures of sodium deoxycholate and oxidized glutathione may be affected by temperature. Methylene violet is released extremely slowly due to the intense electrostatic interactions between the negatively charged nanofibers and the positively charged dye which mediate the hierarchical self-assembly of peptides with surfactants. Sinthuvanich et al. showed two peptide-based gels that were utilized to examine the impact of network electrostatics on chondrocyte behavior using iterative peptide design [92]. At a pH of 7, the MAX8 and HLT2 peptides exhibited formal charge states of +5 and +7, respectively. These peptides were shown to go through environmentally driven folding and self-assembly to produce hydrogel networks with comparable mechanical properties but distinct electropositive characteristics. When triggered with a cell culture medium, HLT2 purified easily, and solutions of unfolded peptide underwent gelation at a rate analogous to MAX8. Additionally, after one hour, the HLT2 gel’s storage modulus (566 ± 76 Pa) was comparable to the MAX8 gel’s storage modulus (763 ± 18 Pa). Further rheological investigations were conducted to compare the shear thinning and recovery characteristics of the two gels. Direct encapsulation and syringe delivery of primary chondrocytes were both possible with each gel. Evaluation of extracellular matrix deposition, cell viability, and shape, as well as the mechanical characteristics of cultivated gel-cell constructions, revealed that network electrostatic affected cell behavior for these peptide-based materials.

Frick et al. have shown peptide-based hydrogels that could be precisely bio-functionalized to produce a suitable substrate to draw neurite outgrowth from spiral ganglion neurons [93]. The bio-functional elements appeared to be crucial for mechanical stability and neurite attachment. These peptide-based hydrogels are potential possibilities for developing a gapless interface between auditory neurons and cochlear implants, since they self-assemble under physiological conditions. Martin et al. showed the preparation of hydrogels based on short amphipathic peptides for the controlled administration of opioids [94]. A new collection of peptide hydrogelators containing β-homo and D-amino acids was developed based on the lead sequence H-FEFQFK-NH_2_, with the main goal of improving the peptides’ proteolytic resistance, which could theoretically allow an extension of the drug release duration. The resulting hydrogels were characterized by dynamic rheometry and cryogenic TEM, and their cytotoxicity was evaluated after self-assembly in aqueous conditions. These peptide-based hydrogels were shown to be effective extended-release devices for the controlled administration of opioids [94]. Garcia et al. showed the first His-containing self-assembling tripeptide without capping groups which, when used to catalyze the hydrolysis of an ester, produced a thermo-reversible hydrogel and served as a simple building-block for soft materials with practical uses [95].

Furthermore, Du et al. performed rheological experiments to reveal that AcNH-Thr-Phe-Phe-CONH_2_ (AcTFFNH_2_) self-assembled to produce a single-cross-linked hydrogel, with the gelation temperature ranging from 8 to 12 °C in a concentration-dependent manner [96]. The stiffness, stability, and self-healing properties of the binary composite hydrogels were especially improved when AcTFFNH_2_ and sodium alginate content levels were 0.5 wt% and 0.3 wt%, respectively. The double-cross-linking and enfolding of sodium alginate in the constructed fibers resulted in a significant improvement in characteristics. Additionally, the hydrogels demonstrated solid-like behavior, a degree of pH and thermodynamic stability, and self-healing capabilities. These qualities were adapted for use in the processing of food.

### 2.1. Protected Peptide-Based Hydrogels

Mahler et al. described the synthesis of a unique self-assembled hydrogel in an aqueous solution with remarkable mechanical stiffness using the Fmoc-Phe-Phe protected dipeptide as a minimalistic building-block [22]. A hydrogel was produced when a highly concentrated Fmoc-Phe-Phe solution was diluted into an aqueous phase. Due to its biocompatibility and rigid 3D structure, the hydrogel could be applied in the field of tissue engineering and regeneration. Furthermore, Jayawarna et al. reported that Fmoc-Phe-Phe could self-assemble into a fibrillar hydrogel under physiological conditions [97]. When using the pH switch approach to analyze a library of seven Fmoc-protected dipeptides comprised of combinations of the four amino acids, namely, glycine, alanine, leucine, and phenylalanine, the authors identified the gelation properties of Fmoc-Phe-Phe at physiological pH. They also demonstrated that Fmoc-Phe-Phe combinations with either Fmoc-Gly-Gly or Fmoc-Lys produced stable gels at physiological pH. According to Shi et al., under aqueous conditions, aromatic moieties such as Fmoc can help peptides self-assemble into hydrogels, since the aromatic groups mediate both π–π stacking and hydrophobic interactions [98]. Najafi et al. showed the self-assembly of Fmoc-Phe-Val under physiological conditions using a “spaghetti-like” pile of an entangled fibrillar network, producing a robust, rigid, and viscous gel-like structure [99]. Since Fmoc-Phe-Val also demonstrated thermo-sensitivity, heating resulted in a stiffer and transparent hydrogel. Furthermore, Zhang et al. demonstrated a new family of hydrogelators based on the Fmoc-^D^Ala-^D^Ala dipeptide [100]. This dipeptide effectively produced hydrogels that reacted to stimuli from ligand–receptor interactions due to the transition taking place upon the binding of the dipeptide to its ligand (i.e., vancomycin). The ability to control the gel-to-sol transition was also established by the same team, as well as showing a library of several dipeptides that have undergone Fmoc modification and showed varying affinities for vancomycin. Furthermore, Fichman et al. described the assembly and characteristics of physical hydrogels produced by the self-association of extremely basic peptide building-blocks [101]. Furthermore, even an N-terminally protected amino acid was shown to function as a powerful hydrogelator which enabled either spatial control of the hydrogel synthesis or manipulation of the gel’s physical properties [101]. The same group also suggested that intermolecular interactions, the order of amino acids, and chirality, as well as hydrophobic, electrostatic, and π–π stacking interactions, play important roles in the mechanism of gel formation.

Orbach et al. have reported nine Fmoc-modified low-molecular-weight peptides, including the Arginyl–Glycyl–Aspartic acid (RGD) motif, which exhibited various supramolecular structures such as spheres, fibers, hydrogel, nanotubes, etc., as well as remarkable physical proprieties [102]. Moreover, the authors showed the fabrication of a biocompatible Fmoc-RGD peptide hydrogel by conjugation with aromatic substituents, which can offer a new way to design cell-adhesive biomedical hydrogels. The authors also showed that the aromatic group played a key role in regulating the self-assembly and physical properties of the resultant hydrogel. Furthermore, supramolecular structure formation was observed with prominent yields, and the stiffness of the hydrogel increased upon the increase of the aromatic groups in the Fmoc-modified peptide backbone, which may be useful in various biomedical applications, such as assembly into three-dimensional networks, tissue regeneration, drug delivery, and tissue engineering [103,104]. Zhang et al. presented the development of advanced peptide-based supramolecular hydrogels mainly consisting of amino acids and nucleobases for applications in drug delivery systems such as ischemia-related tissue and intratumoral, ocular, and subcutaneous administration [105]. The ability to distinguish between normal and pathological cells allows precisely localized therapeutic targeting. These substances could increase cellular retention time, improve permeability, and promote cell internalization with the aid of peptides. Additionally, the peptide’s precise architecture allows for the modulation of drug release profiles as well as a linear, extended release that lasts for more than a few months. They efficiently distribute vaccinations, different cytokines, therapeutic peptides, and small-molecule medications. When employed to administer treatments in intratumoral, subcutaneous, and ischemic tissue, and via ocular delivery, self-assembled peptide-based hydrogel drug delivery systems have demonstrated encouraging results [105].

**Figure 3:**
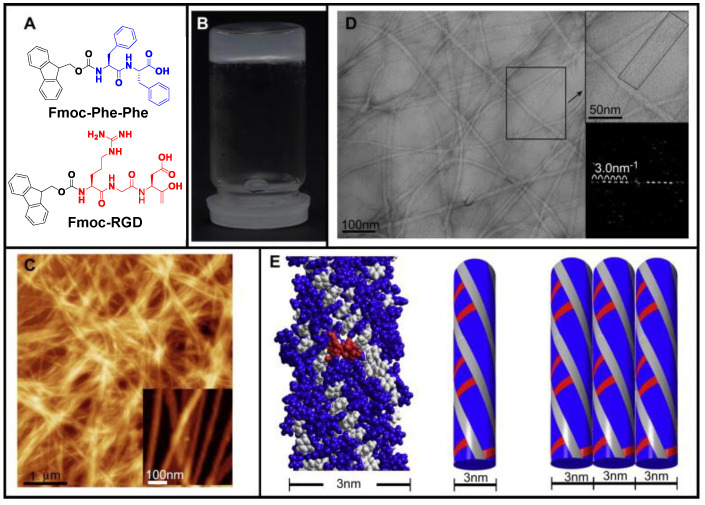
The supramolecular model, nanofibrous structure, and self-assembled hydrogel of Fmoc-Phe-Phe/RGD. (**A**) The chemical structures of Fmoc-Phe-Phe and Fmoc-RGD building blocks. (**B**) At 37 °C, the Fmoc-Phe-Phe and Fmoc-RGD mixture self-assembles into a transparent hydrogel. (**C**) Atomic force microscopy height-imaging of the hydrogel, revealing bundles and entanglements as well as an overlapping mesh of nanofibers. (**D**) TEM imaging reveals the nanofibers to be “flat ribbons,” consisting of small fibrils that are parallel to one another across the width. (**E**) A proposed supramolecular model depicting the lateral assembly of the smaller 3 nm fibrils into larger ribbons. Reproduced with permission from Ref. [107]. Copyright © 2009, Zhou et al., *Biomaterials*, Published by Elsevier Ltd. All rights reserved.

Seow et al. described peptide-based hydrogels, which were further classified into short (≤20 residues) or ultrashort (≤7 residues) peptides that have potential applications in biomedicine [106]. Zhou et al. presented the successful in vitro 3D culture of human dermal fibroblasts using self-assembled bioactive hydrogels composed of Fmoc-Phe-Phe and Fmoc-RGD (Figure 3A) [107]. Fmoc-Phe-Phe (pH 10) and Fmoc-RGD (pH 3) solutions were mixed in various volume ratios to produce transparent Fmoc-Phe-Phe/RGD hydrogels that contained 10 to 50 M% Fmoc-RGD at 37 °C/pH 7.0 in order to obtain hydrogels that were both bioactive and stable under physiological conditions (Figure 3B). The RGD sequences were presented on the fibers’ surface, and the nanofibers conformation was kept stable by hydrogen bonding with nearby peptide sequences (Figure 3C,D). It was further shown that the length of the “flat ribbons” was parallel to regular “diffraction” spots, with a spacing of ~3 nm (Figure 3D inset). The lateral assembly of the 3 nm fibrils into larger ribbons was also observed in the proposed supramolecular model, and the RGD sequences were made more accessible, including bioavailability, by being shown on the fibers’ surface (Figure 3E). These low-cost hydrogel scaffolds for the 3D growth of anchorage-dependent cells have significant potential to enhance the fields of cell treatment, tissue engineering, and basic cell biology. Based on the self-assembled Fmoc-Phe-Phe/RGD hydrogels, it is possible to add other bioactive ligands to these systems to boost the effect of RGD or provide additional bio-functions to meet other biological requirements.

Wang et al. showed that self-assembled peptide-based hydrogels serving as support for mesenchymal stem cell proliferation and differentiation into multiple lineages can be useful in musculoskeletal tissue engineering [45]. Fu et al. showed that, by modifying the peptide sequence and adjusting the synthesis system’s parameters, it is possible to produce self-assembled peptide-based hydrogels with a variety of hierarchical microstructures by a straightforward self-assembling process [108]. The self-assembled peptide-based hydrogels offer numerous practical applications in the biotechnology field, including tissue engineering and drug administration, due to their exceptional biocompatibility and biodegradability. Furthermore, Peressotti et al. showed that self-assembling peptides offer the optimum combination of in situ polymerization with adaptability for bio-functionalization, variable physicochemical properties, and high cytocompatibility [109]. Research gaps in the area of hydrogels for brain regeneration have been highlighted in recent applications of self-assembling peptide materials in neural healing and electrical stimulation therapies. A new class of self-assembled aromatic peptide-based hydrogel nanoparticles with a Fmoc-Phe-Phe core and a D-α-tocopheryl polyethylene glycol succinate (vitamin E-TPGS) monolayer as an outer shell was produced by Ischakov et al. [110] by harnessing a modified inverse emulsion approach and the Fmoc-Phe-Phe building-block’s capacity to produce hydrogels through assembly in water without the requirement for cross-linking or any other covalent bonding. Hydrogel nanoparticles were devolved using several straightforward and repeatable stages. It is believed that the physicochemical properties of the encapsulated molecules that have an impact on their release kinetics determine the encapsulation capacity of the nanoparticles. These hydrogel nanoparticles may be suitable as a prospective drug delivery system. However, additional research is required to assess the potential application of this novel class of peptide-based hydrogel nanoparticles in biomedical applications.

**Figure 4:**
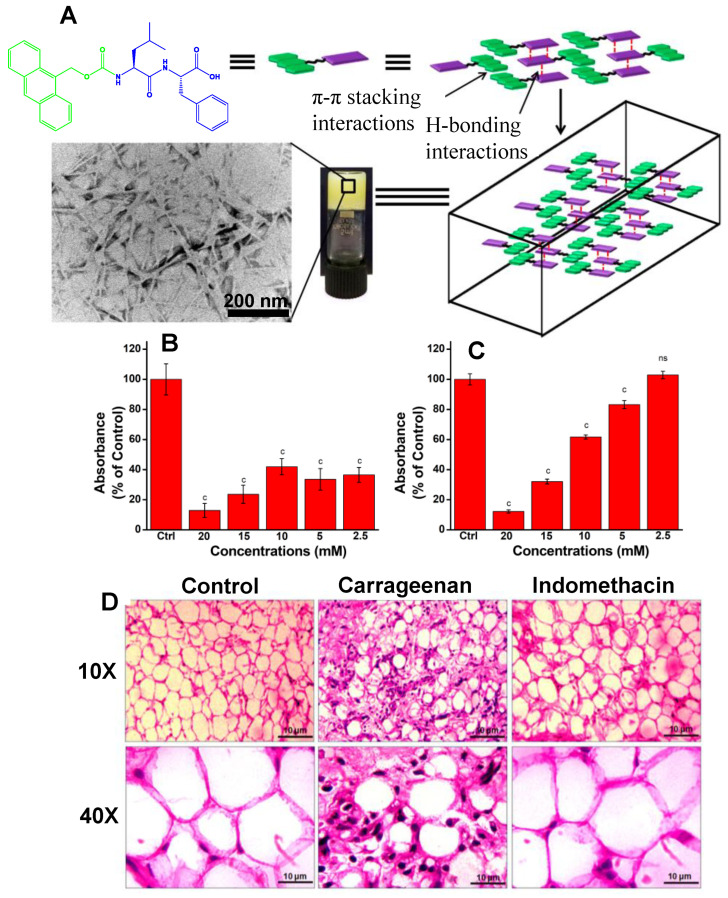
(**A**) Schematic representation showing the formation of a self-supporting hydrogel as a result of the Amoc-capped dipeptide self-assembly. (**B**) Gram-positive and (**C**) Gram-negative bacteria were the targets of the antibacterial experiments. Data are shown as a percentage of the control group. As compared to the control group (*n* = 3), ^c^ *p* = 0.001. An “ns” indicates that the data is non-significant. (**D**) Images of the rat air pouch wall showing endothelial tissue after the studies were stopped at 10× and 40× magnifications. Reproduced with permission from Ref. [111]. Copyright © 2019, Gavel et al., *ACS Appl. Mater. Interfaces*, Published by the American Chemical Society. All rights reserved.

Gavel et al. synthesized a biocompatible, injectable, thixotropic, and self-healing hydrogel based on an Amoc (9-anthracenemethoxycarbonyl)-protected dipeptide [111]. Under physiological conditions (pH 7.4, 37 °C), the peptide trapped a significant amount of water and developed a self-supporting hydrogel through hydrogen bonding and π–π stacking interactions (Figure 4A). Using the rat air pouch model of inflammation, the in vivo compatibility and therapeutic potential of the Amoc-capped peptide hydrogel were examined. The human embryonic kidney cell line HEK293 was used to show that the hydrogels were noncytotoxic in vitro. Gram-positive and Gram-negative bacteria were both effectively combated by the hydrogel’s antibacterial properties. The hydrogel’s antibacterial effectiveness was assessed against Gram-positive and Gram-negative harmful bacteria at the gelation concentration (20 mM) and different additional dilutions (2.5, 5, 10, and 15 mM) (Figure 4B,C). Endothelial tissue histology analyses showed normal tissue architecture in the control group (without the treatments of the carrageenan and indomethacin groups) (Figure 4D). The endothelial tissue in the carrageenan-treated group exhibited edema, which is characterized by widening endothelial cells, a consequent reduction in interstitial gaps, masking of adipocyte size, and the largest infiltration of inflammatory cells such as neutrophils and macrophages (Figure 4D). Carrageenan without additions was administered to the disease control group to cause acute inflammation. In the indomethacin-treated group, a decrease in inflammatory cells was seen that was consistent with the structure of the normal tissue (Figure 4D). 

Chakraborty et al. showed that, by in situ polymerizing aniline (Ani) inside an Fmoc-Phe-Phe hydrogel matrix, a bioinspired, mechanically stiff, and conductive Fmoc-Phe-Phe-PAni hydrogel could be prepared [112]. Additionally, the Fmoc-Phe-Phe-PAni hydrogels are intriguing candidates for use as cardiac cell delivery vehicles, since their capacity for self-healing may enable them to withstand the constant contractions of the heart. Importantly, the authors tested two different applications by adjusting the gelator (Fmoc-Phe-Phe) concentration, which allowed for fine-tuning of the mechanical properties of these hydrogels. Thus, a higher peptide concentration (2% (*w*/*v*)) was utilized for pressure sensing while a lower concentration (0.5% (*w*/*v*)) was employed for cell growth. The Fmoc-Phe-Phe-PAni hydrogels offer a programmable, biocompatible, conductive, and self-healing substrate for biomedical and other technological applications.

Furthermore, a peptide hypergelator containing two Fmoc groups (Fmoc-Lys(Fmoc)-Asp) which self-assembled to form a hydrogel at a very low concentration was produced by Chakraborty et al. [113]. The assembly of the hypergelator involved an unusual two-step method. Studies on the self-assembly of various gelator analogs demonstrate the specificity of the molecular structure required to achieve a very low critical-gelation concentration. Study of a collection of di-Fmoc-based dipeptides generally indicated that these building blocks might function as effective peptide hydrogelators [113]. From an application standpoint, the hydrogel is appropriate for the fabrication of conductive composite hydrogels, based on the integration of conducting polymers, as well as for 2D/3D cell scaffolding. DNA binding with the peptide–PAni composite hydrogel was also demonstrated. Alakpa et al. showed the use of pericytes and mesenchymal stem cells (MSCs) as prospective autologous stem cell sources, and peptide gels with simple chemical composition and tunable physical properties were designed specifically for stem cells to promote targeted differentiation [114]. As a result, an extremely effective bioactive and personalized regeneration scaffold might be found. 

Since short peptides are mostly composed of small recognition modules, they provide an exceptional framework for simulating complex systems and phenomena using straightforward peptide-based model systems. These short peptide-based structures have demonstrated significant potential as materials for adhesives, cell scaffolds, drug delivery systems, antimicrobial agents and surfaces, molecular machinery, and organic-inorganic matrices. Dijk et al. demonstrated that hydrogels composed of a bis-azido peptide and alkyne-functionalized star-shaped poly(ethylene glycol) (PEG) derivatives may be readily fabricated using the Cu(I)-catalyzed 1,3-dipolar cycloaddition procedure [115]. The hydrogels decomposed completely after 40–80 h of incubation under physiological conditions, depending on their cross-linking density, whereas the bis-azido peptide could be hydrolyzed by both plasmin and trypsin. Nanda et al. showed that at physiological pH and temperature, N-terminally protected dipeptides containing a β-amino acid residue produced hydrogels which were used to encapsulate and prolong the release of two vitamins (vitamin B2 and vitamin B12) for a period of three days at 37 °C [116]. Adhikari et al. presented a novel family of hydrogelators based on N-terminally protected artificial self-assembling tripeptides [117]. To investigate how self-assembled tripeptides behave in aqueous media, a sequence of five tripeptides was prepared. At basic pH (11.5–13.5), three of them produced thermo-reversible transparent gels. Additionally, the peptide gelators were very simple to recover by means of altering the medium pH, which can be useful for the removal of dyes from wastewater. Veloso et al. showed the synthesis and investigation of the physical–chemical properties of minimalistic low-molecular-weight hydrogelators such as Cbz-L-Met-*Z*-ΔPhe-OH, Cbz-L-Tyr-Z-ΔPhe-OH, and Cbz-L-Phe-Z-ΔPhe-OH, which formed stable supramolecular hydrogels, whereas the dehydrodipeptides Cbz-L-Ala-Z-ΔPhe-OH and Cbz-L-Gly-Z-ΔPhe-OH did not form a hydrogel [118]. In terms of gelation and rheological properties, the Cbz-protected dehydrodipeptide-based hydrogels showed similar properties and outperformed other low molecular weight peptide-based hydrogels. The rapid, three-step synthesis, along with its scalability and conducive reaction conditions, as well as the accessibility of commercial reagents, make hydrogel design and fabrication readily achievable.

### 2.2. Co-Assembly-Based Peptide Hydrogels

The use of co-assembly methodology was recently established in a variety of applications, including the control of nanostructure physical dimension, the development of non-canonical complex topologies, the modulation of mechanical strength, the design of soft materials that capture light, the fabrication of electrically conducting devices, enzymatic catalysis, induced fluorescence, and tissue engineering [119].

**Figure 5:**
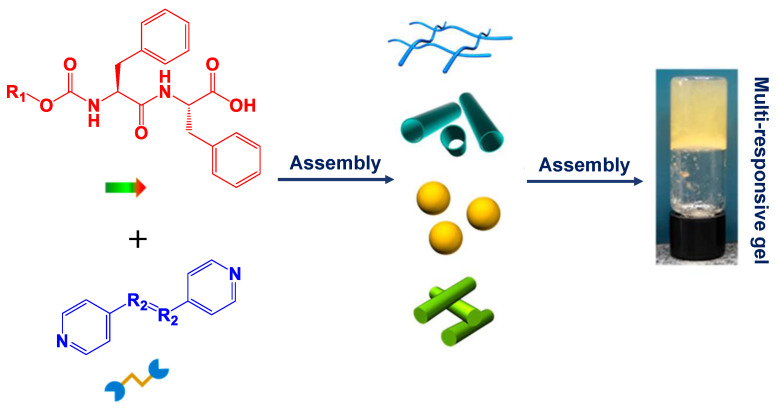
Co-assembly-modulated structural variety of Phe-Phe-based assemblies yielding multiple-responsive gel materials. Hierarchical co-assembly extends the structural diversity and functional range of diphenylalanine-based peptide supramolecular structures. Reproduced with permission from Ref. [120]. Copyright © 2021, Ji et al., *J. Am. Chem. Soc.*, Published by the American Chemical Society. All rights reserved.

Ji et al. developed multi-responsive supramolecular nanofibrils and hydrogels composed of carboxybenzyl-protected diphenylalanine (Cbz-Phe-Phe) and Fmoc-Phe-Phe in the presence of different bipyridine derivatives, mediated by stacking and hydrogen bonding via an aromatic N-terminal group (Figure 5) [120]. By fine-tuning the bipyridine/dipeptide components, the morphological diversity could be readily regulated to produce nanofibrils, nanotubes, nanospheres, and nanorods. The authors showed that the structural transition of Phe-Phe-based peptides into supramolecular structures with various morphologies was induced by a co-assembly approach, and new functional composite gel materials were produced. In contrast, Boc-Phe-Phe and acetyl-diphenylalanine (Ac-Phe-Phe) could only form spheres and crystals in water without forming a gel (Figure 5). This work can serve as a model for the rational design and preparation of multi-component functional supramolecular materials for a variety of future applications.

Ding et al. demonstrated a photo-cross-linking method to increase the mechanical stability of a peptide-based hydrogel by 10^4^, resulting in a storage modulus of ~100 kPa, one of the highest values ever recorded for hydrogels prepared from small peptide building-blocks [121]. The basis of this approach is the light-induced conversion of tyrosine to dityrosine, which is catalyzed by the ruthenium complex. With their significantly increased mechanical stability, photo-cross-linked supramolecular hydrogels are expected to find widespread use in controlled drug release and tissue engineering. Saikia et al. studied the effect of three independent variables that can modify the catalytic activity of self-assembling peptides. A number of catalytic tripeptides were studied to determine the precise contributions of the first two variables, the amino acid sequence and its stereochemistry, to the epitaxial growth and hydrogelation capabilities. They found that the position and chirality of the proline residue control the aromatic π–π stacking interactions that can control the self-assembly of designed peptides and the catalytic characteristics of hydrogels [122]. The third variable, an external electric field, was further investigated to confirm if it had any impact on the catalytic effect of the asymmetric C–C bond-forming aldol process. The amplitude-sweep rheological analysis predicted a fall in the storage modulus of the gels as the field strength increased. The findings demonstrated that a strong electric field could modify the hydrogel’s physical properties, which in turn resulted in the observed variation in enantioselectivity.

**Figure 6:**
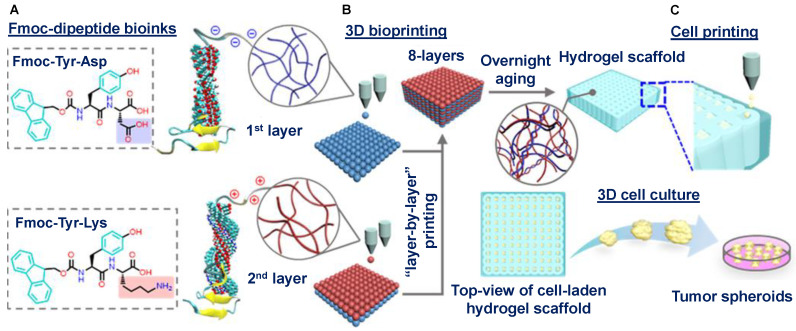
Schematic illustrations of: (**A**) Fmoc-dipeptide-based bioink design, (**B**) 3D bioprinting, and (**C**) the cell culture method for the in vitro formation of tumor spheroid models. Reproduced with permission from Ref. [125]. Copyright © 2019, Jian et al., *ACS Appl. Mater. Interfaces*, Published by the American Chemical Society. All rights reserved.

Lammi et al. showed that by encapsulating the hempseed hydrolysate in ionic self-complementary RADA16 peptide-based hydrogels, an inventive method based on nanomaterials was produced to boost the stability and anti-diabetic characteristics [123]. In various biological experiments, the RADA16-hempseed protein hydrolysate hydrogel was demonstrated as functioning as a new dipeptidyl peptidase IV (DPPIV) inhibitor. The anti-diabetic medication sitagliptin was also administered using this nano-formulation, which helped to lower the drug dosage and subsequent side effects [124]. Jian et al. developed two bioinks, composed of Fmoc-Tyr-Asp and Fmoc-Tyr-Lys, which can self-assemble to form β-sheet amyloid structures (Figure 6A) [125]. Electrostatic interactions between fibers with opposing charges can form Fmoc-dipeptide hydrogels in situ during 3D printing, avoiding the need for post-printing cross-linking with chemicals, ultraviolet light, or enzymes (Figure 6B). Moreover, human hepatoma cells (HepaRG) exhibited more rapid and sustained proliferation in a 3D hydrogel environment compared to 2D petri dish controls in cell culture studies, demonstrating the good cytocompatibility of short peptide-based bioinks (Figure 6C) [125]. 

Firipis et al. demonstrated the improved mechanical properties of Fmoc-FRGDF and Fmoc-DIKVAV to range, while maintaining gelation and fiber formation [126]. These findings considerably increase the adaptability of the Fmoc self-assembling peptides as synthetic biomimetic tissue scaffolds that can be more precisely tailored to simulate certain tissue or cellular microenvironmental requirements, compared to the peptides. Furthermore, Adhikari et al. successfully incorporated reduced graphene oxide (RGO) into peptide-based hydrogels (Fmoc-Tyr-Asp-OH) to produce a stable hybrid hydrogel that contained well-dispersed RGO [127]. In the RGO–peptide hybrid hydrogel, both graphene sheets and gel nanofibers were present. A selected area electron diffraction experiment verified the presence of the RGO sheet and gel fibers. This research shows that RGO can be stabilized within a peptide-based hydrogel system, without the use of any additional stabilizing agents. In comparison to the native hydrogel, the hybrid hydrogel system was stiffer due to the addition of RGO. The development of this type of RGO-containing hydrogel system may allow for a wide range of RGO-based soft hybrid material applications.

## 3. Metabolite-Based Hydrogels

Metabolites are small molecules that are found as intermediate or end products of the metabolic network of various organisms. Key metabolites include amino acids, nucleobases, vitamins, saccharides, and lipids. Recent studies have indicated the ability of metabolites to form supramolecular assemblies.

### 3.1. Nucleobase-Based Hydrogels

Guanine-based hydrogels have been produced since the early 20th century, when it was discovered that 5′-guanosine monophosphate (5′-GMP) can accumulate into polycrystalline gels at millimolar concentrations [128]. The discovery that 5′-GMP can self-assemble into left-handed columnar aggregate particles that can pack into left-handed cholesteric mesophase liquid crystals at higher concentrations is the result of the prospective capacity to alter the properties of these assemblies [129]. Giraud et al. demonstrated the preparation of hydrogels from a new series of hybrid nucleopeptides by incorporating DNA-nucleobases to produce their peptide nucleic acid (PNA) forms [130,131]. Thus, the physicochemical and mechanical properties of the resulting hydrogels can be significantly improved and fine-tuned depending on the type of nucleobase (i.e., thymine, cytosine, adenine, or guanine), with, for example, enhancement of both the gel stiffness (up to 70-fold) and the gel resistance to external stress (up to 40-fold), and the generation of both thermo-reversible and uncommon red-edge excitation shift (REES) phenomena. 

### 3.2. Amino-Acid-Based Hydrogels

Single amino acids have also been shown to construct 3D networks of nanostructures capable of encasing solvent molecules and producing supramolecular gels. Furthermore, the crystalline gel state of L-Phe, the smallest molecule known to date to form gel networks in water, is particularly interesting because of its crystalline gel condition [17,18,132,133]. Zaguri et al. demonstrated the mechanical properties of the Phe single amino acid along with the associated supramolecular fibrillar structures, using scanning electron microscopy and atomic force microscopy [134]. Furthermore, Phe self-assembled fibrils, exhibiting a remarkable Young’s modulus (30 GPa), were comprised of interlaced protofilaments in a helical or twisted ribbon-like supramolecular structure. Furthermore, the crystalline, highly homogeneous, and self-healing Phe hydrogel was formed in H_2_O or PBS at a concentration of 0.24 M following heating at 90 °C and cooling after 5 h, resulting in significantly higher storage and loss moduli compared to other biocompatible fibrillar supramolecular structures. Moreover, Phe could form a hydrogel with high stiffness and nanofibrillar supramolecular structural properties, which may be used in various biomedical and technological applications, such as load-bearing 3D frameworks, self-healing, 3D printing, sensing, and tissue engineering. 

Furthermore, Ramalhete et al. showed that L-Phe hydrogels could be employed as model materials for a nuclear magnetic resonance (NMR)-based analytical method for understanding supramolecular gelation [132]. This method made it possible to pinpoint the additional molecules that changed the material’s characteristics. Amino-acid-containing L-Tryptophan (L-Trp)-based cationic amphiphilic hydrogelators with varied degrees of hydrophobicity were produced by Roy et al. [135]. These hydrogelators showed exceptional bactericidal action against a variety of Gram-positive (MIC = 0.1–75 μg/mL) and Gram-negative (MIC = 0.5–5 μg/mL) bacteria. Nebot et al. provided insight into the aggregation thermodynamics associated with hydrogel formation by molecular gelators derived from L-Val and L-Isoleucine (L-Ile) [136]. Liberato et al. presented His-based hydrogels obtained using photooxidation [137]. The His-based hydrogels were used to produce polymers comprising chondroitin sulfate functionalized with His and recombinant elastin-like peptide (ELP) using a unique mechanism for hydrogel formation via His photooxidation mediated by the singlet oxygen. Furthermore, two new hydrogels were synthesized by Yang et al. through the self-assembly of β-amino acid derivatives [138]. The confirmation of β-amino-acids-based hydrogelators should offer a new way to customize the stability of hydrogels in biological environments and ultimately broaden the ranges of applications of the hydrogels as biomaterials, because β-amino acids are less prone to biodegradation and are therefore expected to be available for longer times. Compared to hydrogels made from α-amino acid derivatives, this type of hydrogel should have a longer bioavailability under biological conditions. Pyrene-conjugated protein amino-acid-based super hydrogels have been shown to form a hydrogel in a variety of aqueous solutions across a wide pH range (7.46–14) [139].

Li et al. developed three-component luminous hydrogels made of melamine, riboflavin, and perylene derivatives functionalized with amino acids [140]. In the hybrid gel systems, hydrogen bonds, triple hydrogen bonds, and π–π stacking interactions form acid-functionalized perylene derivatives. A perylene core and two amino-acid residues were present as the terminal groups, as in the glutamate-functionalized perylene derivatives (GP) and tyrosine-functionalized perylene derivatives (TP). The homogenous self-assembly of the amino acids, together with the fluorescent riboflavin and perylene derivatives resulted in the formation of supramolecular luminescent hydrogel systems. These luminous hydrogels showed exceptional mechanical strength (>104 Pa) and low cell toxicity, which might be used to drive drug release in a regulated manner. Yang et al. showed that, based on self-assembled nanofibers of a β-amino acid derivative, supramolecular hydrogels were enzymatically formed in vitro and in vivo [141]. Juriga et al. evaluated the suitability of various hydrogels composed entirely of amino acids for use in tissue engineering and drug release [142]. Under various circumstances, the impact of the chemical makeup of these hydrogels on their mechanical and swelling capabilities was investigated. Different molar ratios of Lys and Cys were utilized as cross-linkers to produce poly(aspartic acid) (PASP)-based hydrogels. By modifying the chemical structure of the hydrogels using amino acids, PASP-based hydrogels were shown to be promising materials for both medical and pharmaceutical applications. 

As demonstrated by Bratskaya et al., amino acids can also be utilized as chemical effectors to promote a transamination reaction that dissolves salicylimines hydrogels [143]. At pH 8, lysine significantly improved the solubility of N-substituted carboxyethylchitosan (CEC)-salicylimine, reaching 100% at an amino-acid concentration of 20 g/L. Salicylaldehyde’s interaction with carboxy-alkyl-chitosan thus exhibits a new opportunity for the production of biopolymers that are usable over a far wider pH range than previously known for chitosan. Peres et al. showed that using free radical polymerization, pH-responsive hydrogels and nanogels were produced using N-acryloyl-L-glutamic acid (L-AGA) [144]. By using solution homo-polymerization reactions with a monomer concentration of >30% *w*/*v*, physically cross-linked hydrogels were produced. It is interesting to note that less than 20% of the monomer was required to produce fully water-soluble polymers. A hydrophilic drug was successfully loaded into the nanogels, with an encapsulation efficiency of >83% and a drug content of >41 mg_DOX/_g_P_ (mg of doxorubicin (DOX) per gram of polymer). Altogether, these results contribute to the rational design of molecular hydrogelators which could be used for the tailored preparation of this type of soft material for diverse applications.

#### 3.2.1. Composite Amino-Acid-Based Hydrogels

**Figure 7:**
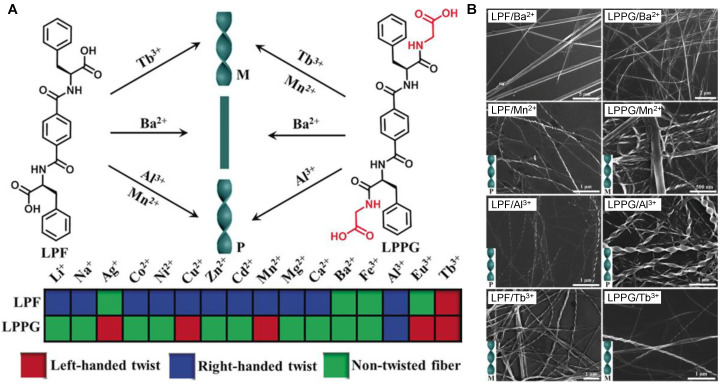
(**A**) Nanostructures are composed of the L-type enantiomers LPF and LPPG and various metal ions. Left- and right-handed chiral nanostructures are designated by M and P, respectively. (**B**) SEM images of LPF/Ba^2+^, LPF/Mn^2+^, LPF/Al^3+^, and LPF/Tb^3+^, as well as LPPG/Ba^2+^, LPPG/Mn^2+^, LPPG/Al^3+^, and LPPG/Tb^3+^, as indicated. Reproduced with permission from Ref. [146]. Copyright © 2018 Wiley-VCH Verlag GmbH & Co. KGaA, Weinheim, Wang et al., *Angewandte Chemie*, John Wiley and Sons. All rights reserved.

Abenojar et al. designed a special thermo-responsive magnetic glycol chitin-based nanocomposite that contained iron oxide nanoparticles and D-amino acids [145]. This material could be delivered and transformed from a solution to a gel state at physiological temperature for sustained release of D-amino acids and magnetic field-activated thermal treatment of targeted infection sites. The D-amino acids in the hydrogel nanocomposite prevented the growth of new biofilms and dislodged those that already existed. Furthermore, loading the hydrogel nanocomposite with magnetic nanoparticles allowed combination thermal therapy after magnetic field stimulation (magnetic hyperthermia); infections resistant to conventional antibiotics were not completely eradicated by separate D-amino acid and magnetic hyperthermia treatments. Using this novel two-step approach which utilized an externally actuated gel-nanocomposite system for thermal treatment after initial disruption with D-amino acids, the authors could demonstrate the complete eradication of the *Staphylococcus aureus* biofilms in vitro. Wang et al. successfully used L-Phe derivatives to self-assemble chiral twisted and non-twisted nanofibers in the presence of different metal ions (Figure 7) [146]. They prepared two L-Phe-based hydrogelators such as LPF and LPPG. Through the coordination interaction between metal ions and supramolecular hydrogelators composed of L-Phe, a simple method was developed to produce the chiral nanotwists (Figure 7A). The inverted test tube method showed that the hydrogels were produced after adding LPF to a variety of metal ions solutions, with the exception of the Eu^3+^ solution (Figure 7B). Except for Li^+^ and Na^+^ complexes, the LPPG complexes with the aforementioned metal ions could also produce hydrogels (Figure 7B). The self-assembled nanostructures’ handedness, twist pitch, and diameter could be easily controlled (Figure 7B) [146]. These chiral twists have tremendous potential in electrochemical sensing, asymmetric catalysis, or chiroptics, and this technology can be used in complementary investigations of controlling the chirality of nanostructures.

Furthermore, Roy et al. showed a self-healing hydrogel composed of amino acids and (11-(4-(pyrene-1-yl)butanamido)undecanoic acid) [147]. Intriguingly, by introducing carbon-based nanomaterials such as graphene, pristine single-walled carbon nanotubes (Pr-SWCNTs), and both graphene and Pr-SWCNTs into the native gel system, it was possible to successfully control the self-healing, thixotropy, and stiffness of the hydrogel. Hybrid gels with RGO and/or Pr-SWCNTs also showed intriguing semiconductive activity.

#### 3.2.2. Fmoc-Protected Amino-Acid-Based Hydrogels

Roy et al. showed that Fmoc-L-Phenylalanine-OH (Fmoc-Phe-OH), an N-terminally Fmoc-protected single amino acid, could produce an effective, stable, and transparent hydrogel at a minimum gelation concentration of 0.1% *w*/*v* [148]. Fluorescent few-atom silver nanoclusters have been produced and stabilized using this hydrogel. Intriguingly, silver ions were complexed with the carboxylate group of the Fmoc-Phe-OH gelator in a water medium in the absence of any hazardous reducing agents. The silver ions were spontaneously reduced at physiological pH and room temperature to produce silver nanoclusters. These chemically stable Ag nanoclusters that emit red light can be used for sensing and other applications. Furthermore, Yang et al. described various Fmoc-modified amino acids, such as Fmoc-Tyr, which were employed as effective hydrogelators, while unmodified amino acids without aromatic capping did not form hydrogels [149]. The group further demonstrated the utilization of a brand-new green Fmoc-Tyr-OH-amygdalin hydrogel for sustained release of amygdalin [150]. This hydrogel exerted neuroprotection by enhancing neurological performance and reducing neuroinflammation. This study emphasizes the extraordinary potential of hydrogel to improve bioactivities. Xie et al. showed that Fmoc-Trp, Fmoc-Methionine (Fmoc-Met), and Fmoc-Tyr were self-assembled to produce transparent, stable self-supporting hydrogels [151]. The antibacterial efficiency of all three forms of hydrogel was excellent against Gram-positive bacteria but minimal against Gram-negative bacteria in the sequence Fmoc-Trp hydrogel > Fmoc-Met hydrogel > Fmoc-Tyr hydrogel. The outstanding antibacterial characteristics of the amino-acid-based antibacterial hydrogels suggest their significant biomedical potential.

#### 3.2.3. Co-Assembly-Based Amino Acid Hydrogels

**Figure 8:**
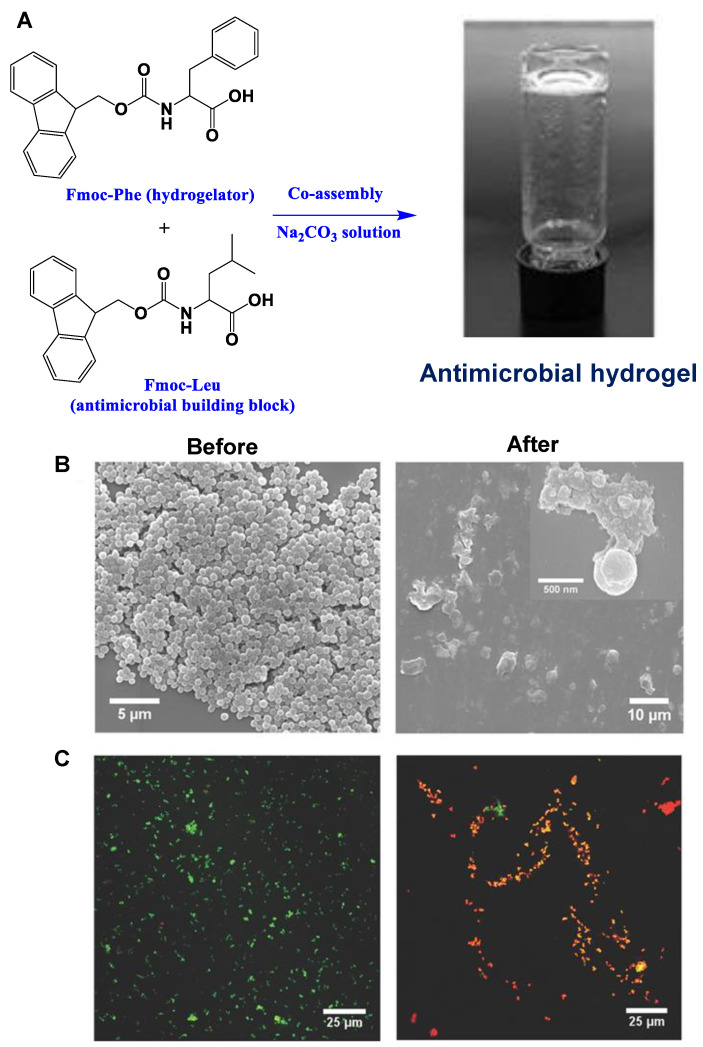
(**A**) Demonstration of the facile preparation of an antimicrobial hydrogel based on the co-assembly of Fmoc-Phe and Fmoc-Leu. (**B**) Representative SEM images and (**C**) overlapping fluorescence images for live/dead bacterial staining assay of *S. aureus* before and after 2-h incubation with the co-assembled Fmoc-Phe + Fmoc-Leu hydrogel. Green color SYTO 9 labeled both live and dead bacteria, while red color propidium iodide stained only dead bacteria. Reproduced with permission from Ref. [153]. Copyright © 2014 WILEY-VCH Verlag GmbH & Co. KGaA, Weinheim, Irwansyah et al., *Advanced Materials*, John Wiley and Sons. All rights reserved.

Croitoriu et al. employed a pH-switch and a polar solvent strategy for the Fmoc-Trp-OH and the Fmoc-Lys-Fmoc-OH stock solution, respectively. In oscillatory shear experiments, stable structures with transparent and solid-like characteristics were produced as a result of the co-assembly of the amino acids in various volumetric ratios [152]. Irwansyah et al. reported a unique supramolecular hydrogel platform formed by Fmoc-Phe which can be easily established by the intermolecular stacking interactions between Fmoc and the phenyl group [153]. Additionally, by combining the Fmoc-Phe hydrogelator with Fmoc-Leu as an antimicrobial building-block, antimicrobial properties could be conferred upon this supramolecular hydrogel platform via a co-assembly method (Figure 8A). While showing biocompatibility towards cultured mammalian cells, the co-assembled (Fmoc-Phe + Fmoc-Leu) supramolecular hydrogel demonstrated selective Gram-positive bactericidal activity through a mechanism involving cell wall and membrane rupture. In contrast to the edges and smooth bodies observed for native bacteria, cellular deformation and surface collapse were detected in *Staphylococcus aureus* cells following a 2-h incubation with the hydrogel (Figure 8B), as also supported by live/dead bacterial staining assay (Figure 8C). In contrast to commonly used antimicrobial hydrogels based on cationic materials, the fabrication of this antimicrobial hydrogel relies on the co-assembly of commercially available Fmoc-amino acids, allowing for the quick and affordable production of a biocompatible antimicrobial hydrogel without the need for time-consuming organic synthesis and purification procedures.

Furthermore, multi-component supramolecular hydrogels have also been reported to form when ε-Fmoc-Lys was combined with either Fmoc-Phe or Fmoc-Leu [154,155]. Guilbaud-Chereau et al. showed that various Fmoc-protected amino acids spontaneously self-assembled into a 3D fibrous network, resulting in the formation of hydrogels [156]. The authors examined binary mixtures of Fmoc-Tyr-OH and Fmoc-Tyr(Bzl)-OH or Fmoc-Phe-OH and Fmoc-Tyr(Bzl)-OH gels. Various microscopy and rheology methods were used to evaluate the structural and physical characteristics of these gels. These hydrogels contained oxidized carbon nanotubes (ox-CNTs) and graphene oxide (GO), which displayed a good interface with the fibrils, particularly the nanotubes. As a high-concentration model hydrophilic drug, L-Ascorbic acid was incorporated into the gels. With the potential for numerous applications, including drug delivery, the heat produced by the carbon nanomaterials upon NIR light irradiation stimulated the rapid release of the drug. Furthermore, Zhao et al. reported a novel conjugated oligomer, oligo(thiophene ethynylene) (OTE)-D-Phe, which was synthesized by adding D-Phenylalanine (D-Phe) to the side chain of conjugated OTE [157]. A novel and biocompatible low-molecular-weight hydrogel (HG-2) was produced through self-assembly by combining Fmoc-L-Phe and OTE-D-Phe. Xing et al. showed smart high-quality hydrogel materials with light irradiation-triggered luminescence using the co-assembly of Phe with bipyridines [158]. Hydrogel obtained from the co-assembly of Phe and bipyridines resulted in fluorescently imprinted materials without showing any photobleaching or phase separation during the irradiation of UV light. Fichman et al. demonstrated the ability to synthesize Fmoc-Tyr, Fmoc-3,4-dihydroxyphenylalanine (Fmoc-DOPA) two-component hydrogels with functional properties of the catechol groups and mechanical properties similar to those of the Fmoc-Tyr gel [159]. Such a multi-component arrangement could potentially enable researchers to take advantage of the Fmoc-DOPA antioxidant activity, radical trapping, and metal chelation functions in addition to its reduction activity. This is an excellent example of how the complementary qualities of different building blocks can be combined to generate new functionalities.

## 4. Conclusions and Future Perspective

In this review, we have described and discussed peptide- and metabolite-based hydrogels including the recent advancements in various biomedical applications such as 3D bioprinting, tissue engineering, antibacterial and wound-healing materials, drug delivery, tumor therapy, water remediation, and other technological applications. Protected motifs, including the carbon composite of peptides and amino acids, provide a facile scaffold for the formation of hydrogels due to the increase in the non-covalent interactions, such as the hydrogen bonding, hydrophobic, electrostatic, and π–π stacking interactions. Furthermore, multi-component-based materials such as carbon composite, co-assembly of peptide, and amino-acid-based multifunctional hydrogels enhance their mechanical properties due to the increase in non-covalent interactions, which can be useful in various technological applications. Moreover, using a minimalistic building-block approach, hydrogel composed of a single amino acid such as Phe exhibited remarkable mechanical properties leading to its applicability in various technologies. 

Taken together, the described studies emphasize the ability of simple bioinspired building-blocks to self-assemble into ordered supramolecular hydrogel networks. Short peptides and metabolites contain all the molecular information needed to direct assembly processes that result in structures that both are biocompatible, due to the identity of the building blocks, and may be utilized for various biomedical applications such as antimicrobial activity and drug delivery. The biomolecular building-blocks could also be part of co-assembled systems that have the desired physical and chemical properties. 

Yet, there are still several challenges that must be overcome in the formation of peptide- and metabolite-based hydrogels, including their design, functionalization, and clinical translation. The biodegradability of peptide hydrogels composed of natural amino acids suggests low scaffold durability and a considerable risk of early drug release or leakage. The use of cutting-edge methods, including 3D printing, will also provide novel composite materials with hierarchical and controlled structures, which may perform better in regenerative medicine and drug administration. Furthermore, it is difficult to characterize hydrogels in vivo and at the cellular level. Real-time monitoring of self-assembly at the cellular level will therefore be a significant advance. Future research is required to examine the effects of adding minimalistic bioactive compounds to the new multifunctional hydrogels. We anticipate that self-assembled peptide hydrogels will have a considerable impact on the effective delivery of therapies in the near future. The design of peptide- and metabolite-based hydrogels with predictable and tunable properties using advanced technologies that can improve scalability, along with new computational methods, is still an unmet need. For upcoming research using the minimalistic building approach, producing sophisticated, multi-functional hydrogels with multi-responsive properties, smart drug release systems and wound monitoring instruments, such as pathogenic infection detectors, could be a promising strategy. Moreover, more research is required to facilitate the rational design of simplistic building-block metabolite-based multifunctional hydrogels with adequate characteristics including multi-responsiveness, low molecular weight, biodegradability, high water content, favorable mechanical properties, biocompatibility, self-healing, synthetic feasibility, low cost, easy design, biological function, and remarkable injectability for various biomedical applications.

## Data Availability

Not applicable.
